# Hurricane impacts on a coral reef soundscape

**DOI:** 10.1371/journal.pone.0244599

**Published:** 2021-02-24

**Authors:** Kayelyn R. Simmons, David B. Eggleston, DelWayne R. Bohnenstiehl

**Affiliations:** 1 Department of Marine, Earth, and Atmospheric Science, North Carolina State University, Raleigh, North Carolina, United States of America; 2 Center for Marine Sciences and Technology, North Carolina State University, Morehead City, North Carolina, United States of America; 3 Center for Geospatial Analytics, North Carolina State University, Raleigh, North Carolina, United States of America; Academia Sinica, TAIWAN

## Abstract

Soundscape ecology is an emerging field in both terrestrial and aquatic ecosystems, and provides a powerful approach for assessing habitat quality and the ecological response of sound-producing species to natural and anthropogenic perturbations. Little is known of how underwater soundscapes respond during and after severe episodic disturbances, such as hurricanes. This study addresses the impacts of Hurricane Irma on the coral reef soundscape at two spur-and-groove fore-reef sites within the Florida Keys USA, using passive acoustic data collected before and during the storm at Western Dry Rocks (WDR) and before, during and after the storm at Eastern Sambo (ESB). As the storm passed, the cumulative acoustic exposure near the seabed at these sites was comparable to a small vessel operating continuously overhead for 1–2 weeks. Before the storm, sound pressure levels (SPLs) showed a distinct pattern of low frequency diel variation and increased high frequency sound during crepuscular periods. The low frequency band was partitioned in two groups representative of soniferous reef fish, whereas the high frequency band represented snapping shrimp sound production. Daily daytime patterns in low-frequency sound production largely persisted in the weeks following the hurricane. Crepuscular sound production by snapping shrimp was maintained post-hurricane with only a small shift (~1.5dB) in the level of daytime vs nighttime sound production for this high frequency band. This study suggests that on short time scales, temporal patterns in the coral reef soundscape were relatively resilient to acoustic energy exposure during the storm, as well as changes in the benthic habitat and environmental conditions resulting from hurricane damage.

## Introduction

Ecosystems throughout the world are increasingly threatened by multiple natural and anthropogenic stressors, often leading to ecosystem shifts from desirable to less desirable states [1–4]. Coral reefs are the some of the most diverse ecosystems on Earth, and the transition between disturbance states is often observed through changes in coral reef community composition and ecosystem function [5–7]. The desired state is an environment that supports critical ecological processes and resulting patterns across space and time, such as overall system production, key predator-prey (or grazer) interactions, and reproduction across multiple functional groups [8, 9]. Disturbance states in coral reef ecosystems are often described as a shift from the desired, structurally complex coral dominated reef, to a less desirable macroalgal dominated state [10–12], as well as reductions in major functional groups such as herbivorous grazers, prey fish stocks, and apex predators [13, 14].

Natural disturbance impacts to coral reef ecosystems vary from chronic events such as coral predation, bioerosion, and intermittent disease, to pulsed catastrophic events such as mass bleaching and hurricanes [15–17]. In certain geographic regions such as the Caribbean, Bahamas and Florida, coral reefs are prone to hurricanes that can cause massive structural damage [18, 19]. Hurricanes can disrupt and reduce ecosystem functions and services [20–24]. Long-term habitat degradation and the persistent decline in the three-dimensional structure of coral reefs can have cascading consequences for reef fish diversity, fisheries, and ecosystem services [25–27]. The response of reef fauna to disturbed benthic habitats can lead to a shift in their spatial distribution as changes in prey densities or habitat specialists compete for sufficient refuge space [28]. Conversely, large-bodied, transient reef fish are more likely to survive an immediate decline in benthic cover because of their ability to relocate to a presumably more desirable habitat [29]. Hurricane impacts have been widely assessed in coral reef ecosystems as catastrophic events that not only promote long-term declines in habitat quality (e.g. algal regime shifts, sedimentation), but further hinder recovery processes from other chronic stressors such as coral disease, overfishing, pollution, and sedimentation [18, 24, 30, 31]. Nevertheless, we know little about how extreme episodic impacts, such as hurricanes, alter the behavior of biological sound production, or biophony, of soniferous (sound-producing) species.

One of the most common quantitative methods to assess the magnitude of change within a population or community pre- and post-disturbance is to assess the temporal response of species composition using abundance or biomass indices [22, 32, 33]. Recent application of passive and active acoustic sampling techniques now allow studies of hurricane impacts to marine organisms using a before, during, and after impact approach [34–36]. For example, acoustic telemetry (active acoustic techniques) of tagged fish characterized the success or failure of nocturnal foraging reef fish (e.g. grunts, snapper) to find refugia, as well as their vulnerability to predation, following a severe disturbance [37]. A recent study on juvenile bull shark (*Carcharchinus leucas*) movements before and after hurricane Irma also described predatory behavioral responses related to shifting prey densities [38]. Although active acoustic studies provide information on individual animal movements, passive acoustic monitoring provides information on soniferous species assemblages that use sounds to communicate, and thereby can be sampled to reflect potential deviations in behavior in response to disturbances such as hurricanes [39–41].

### Soundscape ecology

Soundscapes, the collection of biological, environmental, and anthropogenic sound sources within an ecosystem, can provide high resolution spatiotemporal information about ecosystem patterns and processes [42–44]. Critical information about habitat-specific biodiversity and environmental conditions can be derived from passive acoustic monitoring [44, 45]. Additionally, soundscape analysis allows for the passive acquisition of species assemblage patterns without the influence of human interactions [46–48]. The presence of divers can alter fish distribution and behavior as a negative (i.e. avoidance) or positive (i.e. aggregate) association with human presence [49–51]. Soundscapes provide a collection of empirical data in a natural state without disrupting critical biological or ecological interactions, and allows for visualization of various temporal patterns in acoustic activity and inferred behavior (e.g. hourly, daily, annually, seasonally) for soniferous species. The ecological application of soundscapes is becoming more widely accepted as an indicator of species presence/absence, habitat associations, and complex biological interactions (e.g. territorial behavior, spawning aggregations, migratory patterns) with applications across a wide range of terrestrial (e.g. woodland forest, desert) [52–54] and aquatic (e.g. coral reefs, oyster reefs, seagrass beds, kelp forest) [55–59] ecosystems.

### Application of soundscape ecology to disturbance impacts

Soundscape methods have been useful in distinguishing between healthy and degraded ecosystems largely by recording the presence and absence, as well as behavior of key soniferous taxa [44]. For example, distinct changes in important ecological behavior (e.g. foraging, mating) or daily activities across space and time can reflect noise avoidance or acoustic masking [60–62], with the former resulting in quieter areas due to a decrease in soniferous species abundance and diversity [63, 64]. Landscape ecology studies are increasingly relying on bioacoustic monitoring to assess deleterious impacts resulting from human land use activities (e.g. clear cutting, forest fire, habitat destruction, noise pollution etc.), and to assess changes in biodiversity, spatial distribution, and animal behavior [65–67]. In a broader context, soundscape studies in terrestrial systems are proving to be instrumental in rapidly assessing biodiversity and informing management recommendations for ecological conservation in the aftermath of detrimental anthropogenic and natural disturbances [68, 69]. Thus, understanding the interaction between disturbance states due to natural or anthropogenic impacts and changes in a soundscape are becoming increasingly relevant to management in terrestrial [70, 71] and aquatic ecosystems [72, 73].

Underwater soundscape monitoring is unique in its access to sound-producing invertebrates and resident reef fish assemblages. In underwater environments, sound is an important indicator of habitat quality [74–76], where relatively high densities of soniferous species may indicate high ecosystem health or structural complexity via an abundance of refugia [77]. For example, Freeman & Freeman [78] used coral reef soundscapes to quantitatively assess the correlation between dominant biological frequencies and habitat quality, in which macroalgal dominated reefs are an indicator of reef degradation and were dominated by high frequency sounds produced by benthic invertebrates. Underwater soundscape studies have been successful in characterizing critical spawning habitats, estimating soniferous species abundance, and characterizing community-level interactions [79–81] by collecting semi-continuous, non-invasive information when traditional sampling methods, such as use of nets or diver surveys, are logistically not feasible (e.g. at night, during extreme storm events). Recent work on coral reef soundscapes have provided baseline data on spatiotemporal variation of coral reef soundscapes across various disturbances states such as dead coral cover, high crustose coralline algae cover, and other degraded habitats [82–85].

The presence of rainfall, wind and wave activity on the ocean’s ambient soundscape is well established [86, 87]; however, few studies have focused specifically on modification in the soundscape during extreme weather events, such as hurricanes. Weather and climate may also indirectly influence abiotic sound production by controlling the distribution of ice at high latitudes [88], with the collapse of large ice sheets in Antarctica elevating sound levels throughout the southern Pacific and Indian Oceans [89]. In some ocean basins, the soundscape may be disrupted by large earthquakes that generate high amplitude sounds over time scales of minutes, or by intense episodes of submarine volcanism, which may extend for periods of days-to-weeks [90–93]. The potential ecological significance of these transient natural sound sources is not well understood; however, like hurricanes, they dominant the low-frequency portions of the acoustic spectrum that can be critical in the communication of many marine fauna.

### Hurricane Irma and objectives

On September 2017, Hurricane Irma (Category 4) traveled across the Lower Florida Keys with sustained hurricane force winds (>64kts) extending 130 km from the center [94]. Hurricane Irma passed directly over the Florida Keys National Marine Sanctuary (FKNMS) nearshore marine habitats before making landfall near Cudjoe Key, Florida (USA) [94, 95]. Short-term impacts by large freshwater inflows resulted in changes in the phytoplankton community in nearby coastal canals, with phytoplankton communities returning to normal seasonal patterns within 3 months after the hurricane [96]. The impacts to the Lower Florida Keys seagrass communities from Irma were generally localized, with species-specific beds of seagrass uprooted, and loss of seagrass from storm water runoff resulting in low dissolved oxygen and persistent hyposalinity, similar to historical datasets [97, 98]. Coral reefs in the Middle and Upper Keys showed a significant decline in abundance of the keystone urchin grazer *Diadema antillarum*, as well as loss of sponges and hydrocorals due to high sedimentation [99].

**Fig 1:**
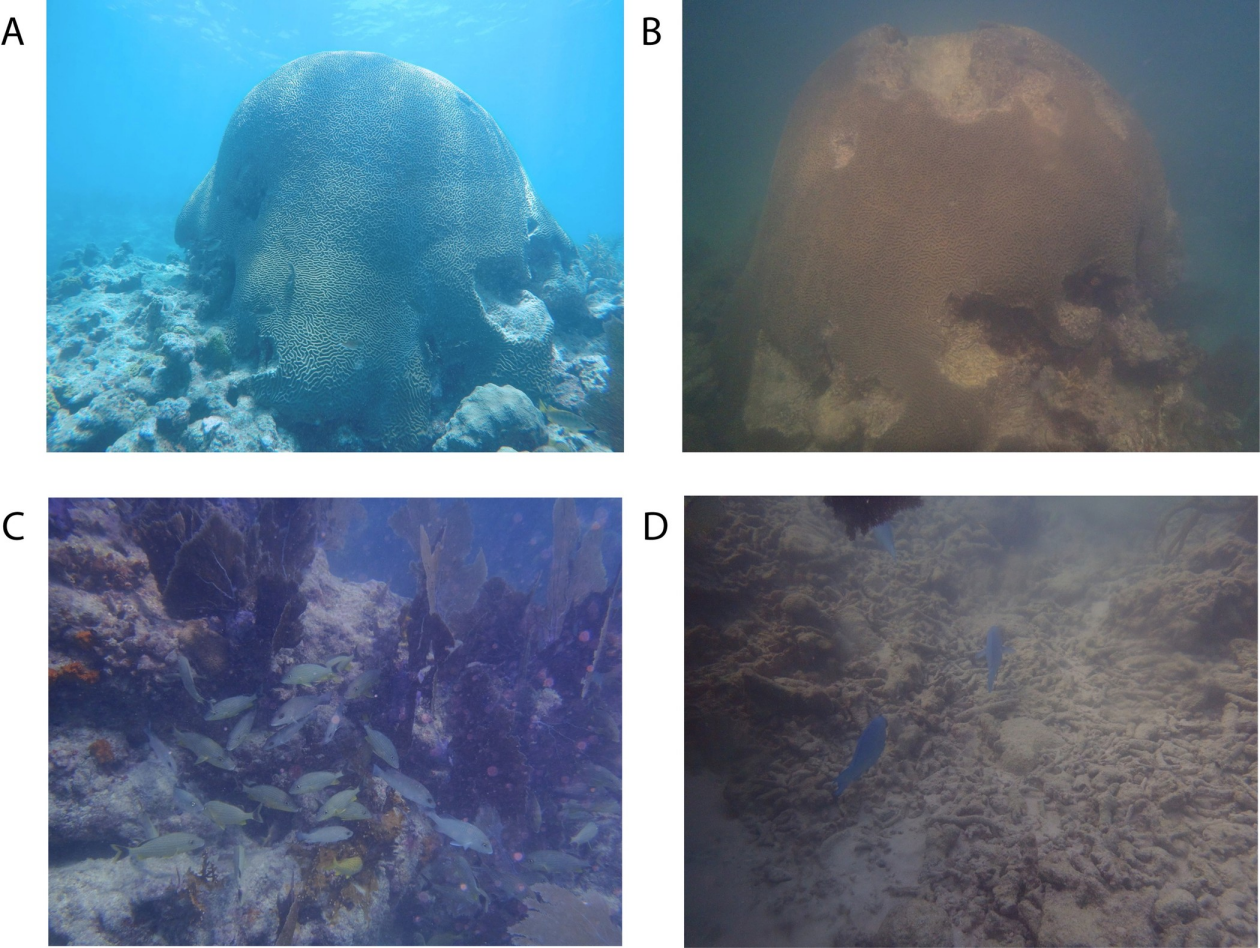
Before-after impacts of hurricane Irma on coral reefs in the Florida Keys, with before images taken in August 2017 and after images taken in December 2017. (A) Brain coral at Eastern Sambo study site taken in August 2017 and (B) its structural damage after Irma in December 2017. (C) Divers observed fish aggregations near and underneath collapsed reef ledges at Looe Key reef (~26 km northeast of Eastern Sambo study site), and the (D) same site with high amounts of reef rubble after Irma. Photo credit K. Simmons.

During October 2017, NOAA science divers and partners surveyed more than 50 coral reef sites from Biscayne Bay (near Miami) to the Marquesas (southwest of Key West) and described severe damage in the Middle and Lower Florida Keys sponge and coral communities from storm force waves, fast-moving debris, and heavy sediment deposits [100]. Sedimentation was the most common impact among sites, resulting in poor visibility and high amounts of marine debris [100]. In December 2017, NC State science divers surveyed eight fore-reef sites, including ESB and WDR, and observed poor visibility (<3m), loose rubble, collapsed reef ledges with a mix of schooling species, as well as sedimented and fragmented sub-massive reef-building corals (Fig 1, personal observation K. Simmons). The short-term disturbance in environmental conditions and the remaining fractured reef habitat structure likely impacted marine faunal interactions and behavior; however, little is known about how these changes in the coral reef habitat are reflected in the sound production of coral reef animals that are mobile.

Passive acoustic recordings were used to characterize the underwater soundscape of the coral reef tract in the lower Florida Keys, USA before, during and after Hurricane Irma. In the weeks following the storm, the biological sounds produced by fish exhibited similar pre-disturbance temporal patterns, and the high frequency noise associated with snapping shrimp showed only a small shift in its diurnal patterns. This opportunistic study investigates the utility of soundscapes in assessing disturbance impacts to the coral reef soundscape generated by soniferous reef fishes and snapping shrimp within a track of the Florida Keys reef system impacted by Hurricane Irma. The main objectives of this study were to (i) quantify the cumulative acoustic exposure associated with the passage of hurricane Irma, and (ii) identify and quantify temporal changes within the biophony in response to Irma with emphasis on daily and diurnal soundscape patterns.

## Materials and methods

### Study system

**Fig 2:**
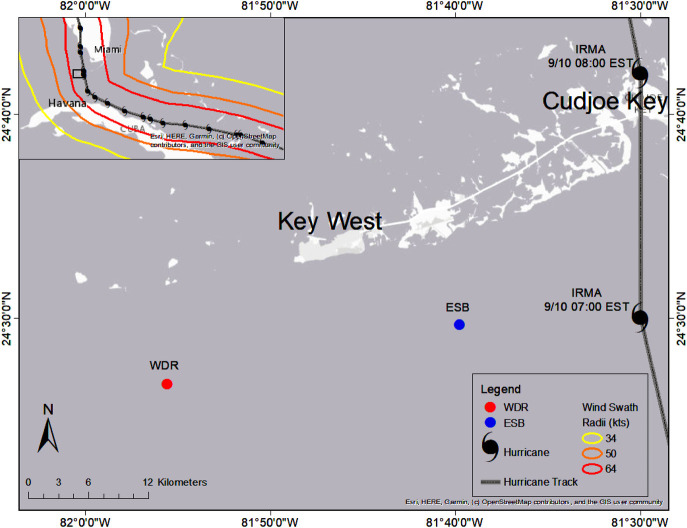
Study site–FKNMS Zone D. Sites are denoted by initials: Western Dry Rocks (WDR) is shown in red and Eastern Sambo (ESB) is shown in blue. NOAA wind swath data are shown as contour lines for maximum sustained wind speeds for 2mins/10meters at 34kts (yellow), 50kts (orange), and 64kts (red). Credit: NOAA NHC Best Track Data updated 06/30/2018.

Underwater soundscape characterization was conducted within the lower (Zone D) FKNMS, which comprises a network of marine reserve types and regulated fishing habitats designated in 1990 [101] (Fig 2). This region is part of the Florida Keys Coral Reef Tract, a large bank-barrier reef system that extends 350 km from the Florida Straits northward to St. Lucie Inlet, Martin County [102]. The lower FKNMS habitat includes a mosaic of shallow, marginal reef systems with spur-and-groove reef formations, reef rubble and a diverse array of hardbottom habitat (e.g. stony corals, soft corals, sponges, macroalgae, adjacent seagrass beds). No-take, marine reserves within the FKNMS vary in size, yet most are relatively small (~0.2 to 0.5km^2^). The Lower Keys often have higher salinity and turbidity relative to the Middle and Upper Keys region due to nearshore transport of nutrient-rich deep water [103] facilitated by the Florida Current, gyre system [104, 105].

As a part of a larger study by our research group, eight hydrophones were deployed in July 2017 across several marine reserve zones. The research was permitted by the Florida Keys National Marine Sanctuary Program (FKNMS-2016-111-A1) and the National Wildlife Service (NW5-SAJ-2016-02423). After the passage of Hurricane Irma (Category 4) in the lower Florida Keys on September 2017, only 2 of 8 hydrophones were recovered: (1) Eastern Sambo, a no-entry reserve, and (2) Western Dry Rocks, which is open to fishing (Fig 2). The other hydrophones were lost, presumably due to wave action and surge from the hurricane. The hydrophone at Western Dry Rocks was recovered after the hurricane lying in sand near the mooring, which removed our ability to use these data to quantitatively assess the post-disturbance soundscape. Western Dry Rocks (WDR—24.445°N, 81.926°W) is a regulated fishing site ~22 km southeast of Key West, FL within the FKNMS. This reef is characterized by wide spur-and-groove sand channels with high relief ledges and a mean depth of 6m. Although live coral cover is relatively low compared to protected reefs, the benthos consists of gorgonians, coral rubble, and hard-bottom substrate. Eastern Sambo (ESB—24.491°N, 81.664°W) is one of four Special-Use Areas (SUA) or no-entry/no-take zones within the FKNMS, and is no-access except for permitted scientific research, restoration, monitoring, or educational purposes. This reef is characterized as a spur-and-groove bank reef with a mean depth of 5m comprised of massive reef building corals, sponges, and gorgonians.

### Environmental data collection

Hurricane Irma’s track, wind swath and landfall data were accessed from NOAA’s National Hurricane Center report on Irma (https://www.nhc.noaa.gov/data/tcr/AL112017_Irma.pdf). Hurricane Irma made landfall near Cudjoe Key in the lower Florida Keys at 08:00 Eastern Standard Time (EST) on September 10, 2017 (Fig 2) before continuing north toward central Florida. Maximum wind speeds reached 115kts with a minimum barometric pressure of 931 hPa at landfall. Wind swath radii were defined as maximum sustained 1-minute wind speed values for tropical storm force winds (34kts), storm force winds (50kts) and hurricane force winds (64kts).

Barometric pressure data were used, independent of the acoustic time series, to delineate the passage of the storm over the reef. Storm duration was defined as the time window over which the pressure fell and remained below its 2.5% quantile level for data collected between July and October 2017. Barometric pressure data were obtained from the Sand Key Lighthouse, Buoy Station ID SANF1 (24.456°N, 81.877°W), located ~ 5km from WDR. These data were recorded hourly with a standard barometer elevation at 14.6 m above the mean sea level.

Because underwater acoustic time series are non-stationary (i.e., have time dependent mean and variance), soundscape changes must be evaluated within time windows before and after the storm that minimize the effect of processes, such as lunar phase (e.g., [85, 106, 107]), that are likely to influence biological sound production on relevant timescales. To account for this potential influence, 18-day and 24-day periods spanning the same portion of the lunar cycle around the New Moon were identified before (New Moon on August 21st) and after (New Moon on September 20th) the hurricane. These time periods were constrained by the timing of the storm and length of our acoustic time series. Astronomical data were obtained from the US Naval Observatory Portal (www.usno.navy.mil/USNO).

### Acoustic data collection and analysis

The coral reef soundscape was monitored via bottom-mounted hydrophones (Soundtrap ST300, Ocean Instruments NZ) suspended ~0.15m above the sandy bottom of the fore-reef at each reef site. Both hydrophones began recording on 14 July 2017 and ended on 01 October 2017 (WDR) and 17 October 2017 (ESB). Both recorders were recovered by a dive team on December 2017. The WDR hydrophone was found lying flat in the sand and detached from the mooring. A spectrogram of the WDR data (Fig 5) indicates a change in acoustic coupling after the hydrophone came into contact with the seabed. Although fish chorusing and snapping shrimp activity are still evident in the time series, the post-storm WDR data were excluded from our quantitative before-after comparisons of the soundscape.

The hydrophone recorders were calibrated with a flat frequency response over the ~0.02-40kHz band. Hydrophones were programmed to record 2 minutes of acoustic data every 20 minutes (72 files/day) with 16-bit A/D conversion and at a sample rate of 48kHz. Acoustic recordings were processed in MATLAB using purpose-written code. The mean spectrum of the acoustic data was calculated for each 2-minute recording using the fast Fourier transform, with a window length (NFFT) of 2^14^ samples and a frequency resolution (Δ*f*) of 2.93 Hz. Hydrophones were also equipped with a temperature sensor that recorded once during each acoustic sampling period.

Fish sounds occupy the low-frequency spectrum (<50Hz to several kHz), often competing with background environmental noise (i.e. wind, wave action) in similar frequency bands [86, 108]. Sound Pressure Levels (SPLs) were calculated at several frequency bands of ecological interest: (1) a low frequency band L1 (50-300Hz) representative of the fish families Serranidae [109–111], Holocentridae [112], and Pomacentridae [113], (2) a low frequency band L2 (1.2–1.8kHz) representative of Haemulidae [114, 115], Lutjanidae [116], Scaridae [81], Sciaenidae [116, 117], and (3) a high frequency band H (7-20kHz) representative of snapping shrimp (Alpheidae), which are a dominant sound producer in coral reef habitats [82, 83, 118, 119]. See S1 Fig for examples of fish calls in each representative band.

To minimize the potential influence of anthropogenic noise and “fish bump” signals in the acoustic time series, SPL data before and after the storm were trimmed to exclude files constituting the loudest 2% of the data over these combined intervals (S2A Fig). Incidental fish bumps are transient signals caused by the physical interaction of an animal with the hydrophone or hydrophone mooring, generating artifacts in the acoustic data [120–122]. Trimming excludes those files containing large amplitude fish bumps (S2B and S2C Fig), as well as files with anomalously large SPLs due to transient boat noise (S2D Fig). For a given recording window (i.e. 00:00, 00:20, ….23:40), the trimmed mean SPLs before and after the storm were calculated for each band (L1, L2, and H). Uncertainty (68% confidence interval) was estimated using a bootstrap resampling procedure (see below).

Generally, for reefs in the South Atlantic and Caribbean, as well as in other coral reef systems, biologic sound production varies diurnally. These patterns often reflect the abundance or acoustic behavior of multiple species, with times of peak acoustic activity in a given frequency band varying between reef systems [e.g., 57, 83, 106, 123, 124]. Because these daily acoustic patterns tend to persist, even as average SPLs may rise and fall, the disruption of this pattern following a disturbance event may indicate changes in the abundance or acoustic behavior of the impacted species. We therefore investigated the daily SPL patterns (over the 72 recordings made each day), as well as the diurnal (daytime vs. nighttime) differences in SPLs within each of the ecologically relevant frequency bands. The decibel difference between daytime and nighttime SPL provides way to normalize for the non-stationarity of the acoustic time series on longer time scales—as opposed to making inferences based on small changes in the absolute SPL before and after the storm. Daily and diurnal patterns are absent or masked during the storm, and therefore not discussed.

For each 24-hour period, daytime and nighttime mean SPLs were also calculated from the trimmed SPL time series. Daytime was defined as the period between local sunrise (05:48–06:19 EST) and sunset (18:15–19:18 EST), whereas nighttime was defined as the period between sunset and sunrise. Uncertainties (68% confidence interval) in the means were estimated using a bootstrap resampling (5000 draws). The probability that the mean daytime SPL was higher than the mean nighttime SPL on each day was estimated from the portion of resampled outcomes with ${\overset{-}{SPL}}_{day}>\;{\overset{-}{SPL}}_{night}$. Values of *p* ≈ 1 indicate significantly higher daytime sound levels, and values of *p* ≈ 0 indicate significantly higher nighttime sound levels on a given day.

For the 18- and 24-day windows assessed before and after the hurricane, the mean difference between daytime and nighttime SPLs, and the confidence intervals for this difference, were estimated using a paired resampling (5000 draws) of the nighttime and daytime means for each 24-hour period. The probability that daytime SPL was greater than nighttime SPL was calculated from the resampled differences, where *p* ≈ 1 indicates significantly higher daytime sound levels, and *p* ≈ 0 indicates significantly higher nighttime sound levels over the assessment window.

### Hurricane acoustic energy exposure

Hurricanes represent broadly distributed acoustic sources, with the sounds recorded at each hydrophone arriving from a range of azimuths and incidence angles. However, to place the acoustic exposure at these reef sites in context and make comparisons with other sound sources, we quantified the acoustic exposure by representing all storm related noise as being sourced from a point at the sea surface directly above each hydrophone and calculating the equivalent energy.

Over the four-day duration of the storm, the received root mean square SPLs calculated for each file were corrected to acoustic source levels (referenced @ 1m) assuming spherical spreading loss between the sea surface and seafloor. The equivalent acoustic power (J/s) that radiated into the water column (i.e., across a 1 m radius hemisphere with surface area 2*π*) was then estimated assuming a constant water density (1030 kg/m^3)^ and sound velocity (1485 m/s) [125]. The acoustic energy was determined by integrating these power values over the duration of the storm, assuming each two minute file is representative of a surrounding 20 minute time window, and then subtracting the energy that would be calculated if the procedure was repeated using the mean background (pre-storm) noise levels. This energy exposure value can then be compared to the equivalent energy that would be associated with common natural and anthropogenic sources (e.g. fishing vessels) operating over a set duration (e.g., [126, 127]) if these sources were fixed in position at the sea surface directly above the hydrophone. This value, however, does not represent the total acoustic energy imparted by the storm.

## Results

### Environmental conditions

**Fig 3:**
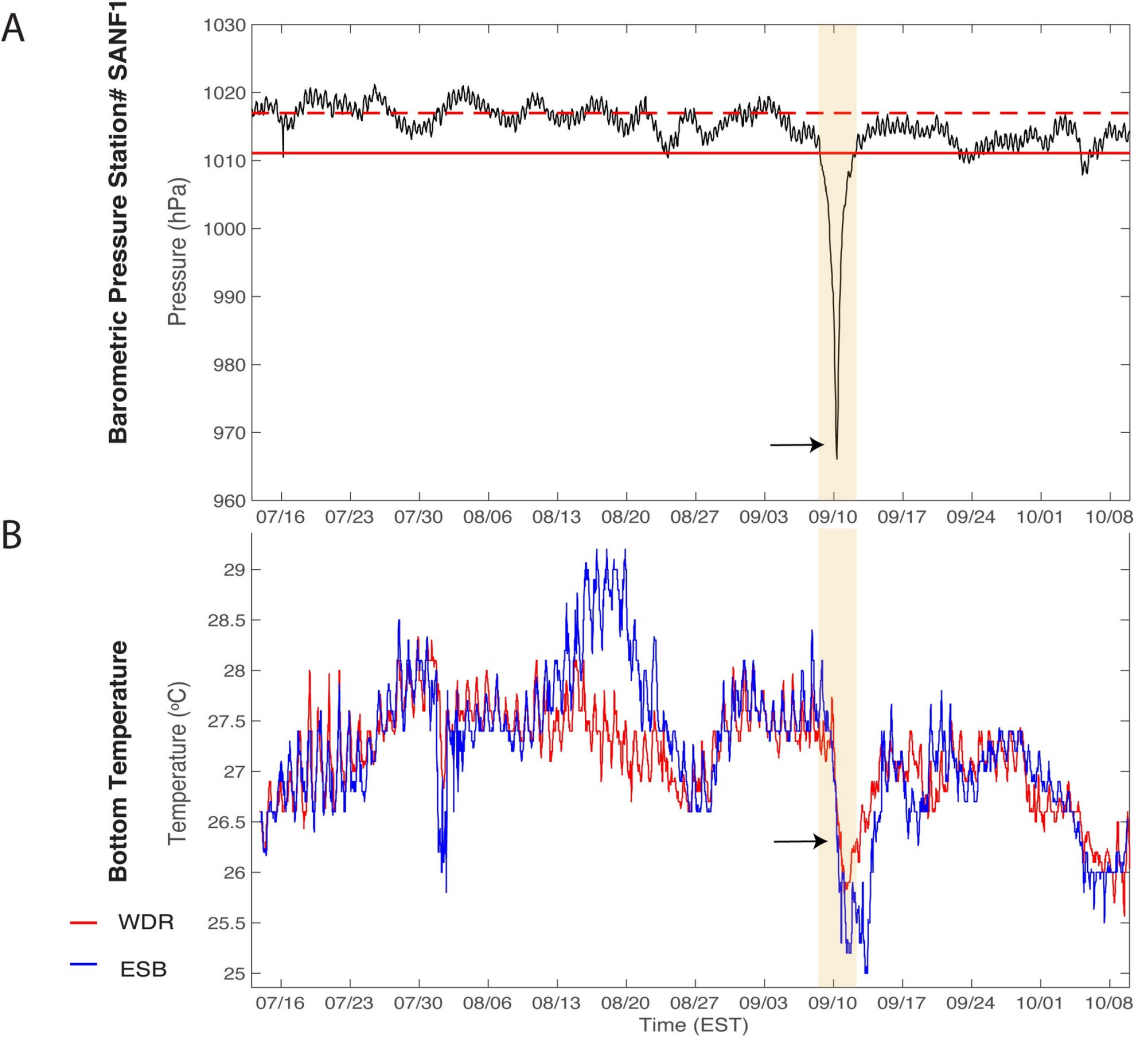
Environmental data. (A) Barometric pressure data from Sand Key Lighthouse, FL (Station ID SANF1 24.456°N, 81.877°W) NOAA-National Data Buoy Center is shown as a black line with the median (dashed red) and the lower 2.5% confidence interval (solid red). (B) Mean hourly bottom temperature (°C) from hydrophone sensor for Western Dry Rocks (red) and Eastern Sambo (blue). The orange bar represents Hurricane Irma duration and the black arrow indicates the times of landfall at Cudjoe Key FL on September 10, 2017 08:00 EST.

Barometric pressure data exhibited semidiurnal oscillations characteristic of the Florida Keys region (Fig 3A). The passage of the storm is marked by a period of low (< 1011 hPa) barometric pressure, which extends from ~12:00 on September 8, 2017 to ~12:00 September 12, 2017 (4 days), reaching a trough at 966 hPa on September 10, 2017 at 06:50 (all times EST). In analyzing the soundscape during the pre- and post-storm windows, a 1-day buffer was applied on either side of the hurricane, such that the pre-storm period ends on September 7^th^ at 12:00 and the post-storm period begins on September 13^th^ at 12:00.

Before and after the storm, daily bottom temperatures at WDR and ESB varied between 26–28°C, except for a short period of slightly increased temperatures at ESB between August 15 to August 19, 2017, which was likely influenced by the lunar spring tide. Both sites exhibited a sharp decline in bottom temperature reaching 25°C shortly after the hurricane made landfall. (Fig 3B). Post-hurricane, cooler water temperatures remained a few days longer at ESB than WDR before returning to pre-disturbance daily temperature oscillations.

### Acoustic spectra

**Fig 4:**
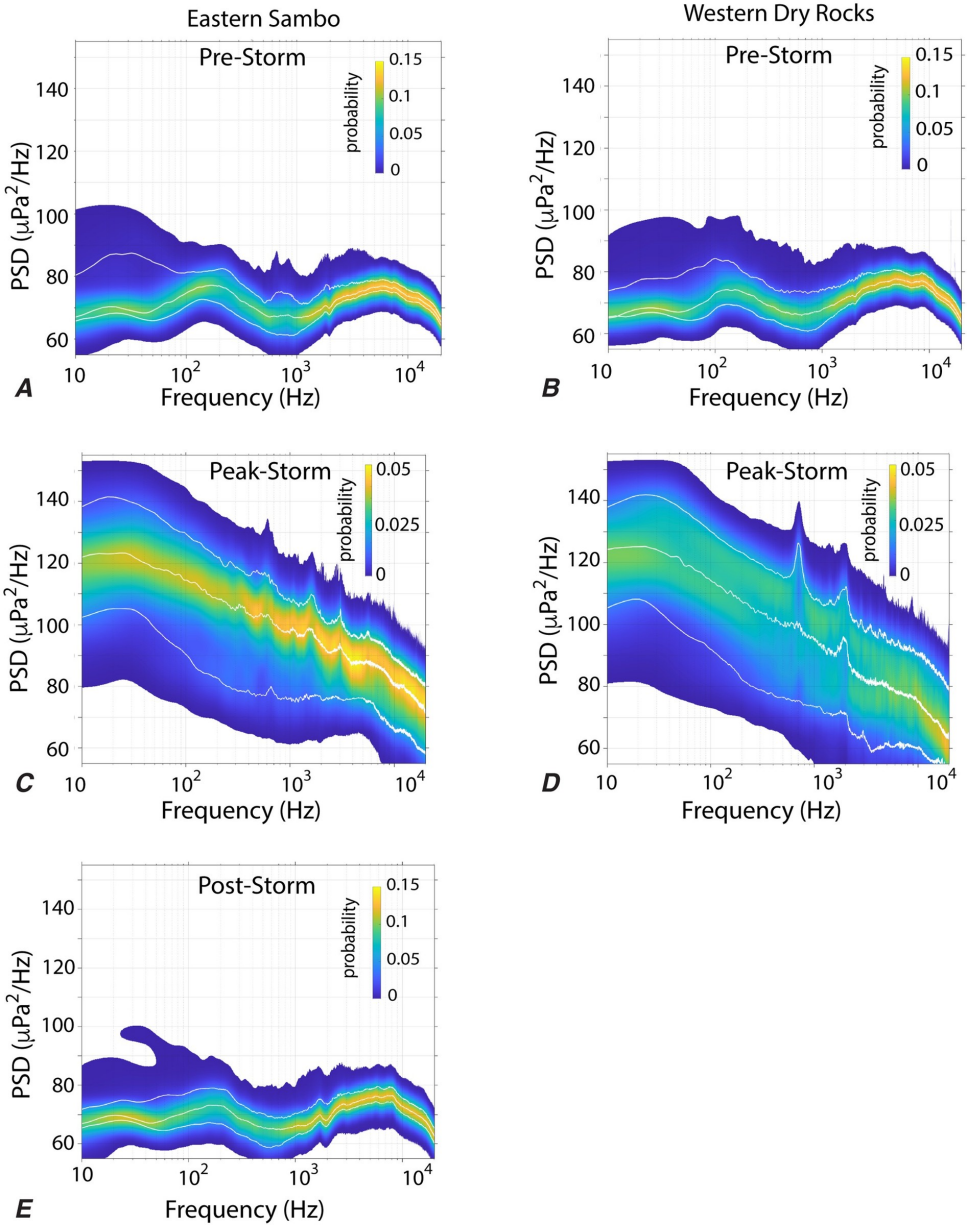
Power Spectral Density (PSD) plot. Power spectral density plot of Eastern Sambo (left) and Western Dry Rocks (right) pre-storm (A, B), peak-storm (C, D), and post-storm (E). The colors show the probability distribution of the spectral amplitudes, and white lines show the 5, 50, and 95% quantiles of power spectral density as a function of frequency.

The acoustic spectra were assessed over the peak of the storm period (September 9^th^– 10^th^) and compared with the spectra over four-day periods immediately before (September 3^rd^– 7^th^) and after (September 13^th^– 17^th^) the storm (Fig 3). Over the four days before the hurricane, the spectra at each site was elevated broadly across the 50–300 Hz frequency range, with additional low amplitude spectral peaks in the frequency ranges of 600-900Hz and 1600-1900Hz being observed most clearly at ESB (Fig 4A). During the peak of the hurricane, the low frequency component of the soundscape was impacted most dramatically and median spectral power increased by 40–50 dB over pre-storm levels in the ~10–100 Hz frequency range, and with multiple narrow band spectral peaks at frequencies of 100’s to 1000’s Hz observed at both sites (Fig 4C and 4D). Within the four-day window after the hurricane, spectra at ESB remained elevated in the 50–300 Hz and 1600-1900Hz frequencies (Fig 4E), yet the pre-storm peak between 600-900Hz was absent.

### Acoustic energy exposure

WDR experienced a higher cumulative energy exposure than ESB estimated at 9.9 x 10^3^ J and 4.8 x 10^3^ J, respectively. In comparison to other acoustic energy disturbances commonly experienced in the lower Florida Keys region, the exposure over the duration of Hurricane Irma was comparable to small vessel (SL = 153 dB re 1μPa @ 1m) operating continuously [128–130] directly overhead for 1 week (ESB) to 2 weeks (WDR). The WDR hydrophone presumably detached from its mooring at some point during the storm; however, the exact timing of this event was not readily identifiable, and no corrections were made to account for potential changes in sensitivity of the instrument. Estimates of acoustic exposure also do not account for the signals produced by debris impacting the hydrophone and mooring, since this effect is not easily disentangled from the acoustic wavefield.

### Biophony

**Fig 5:**
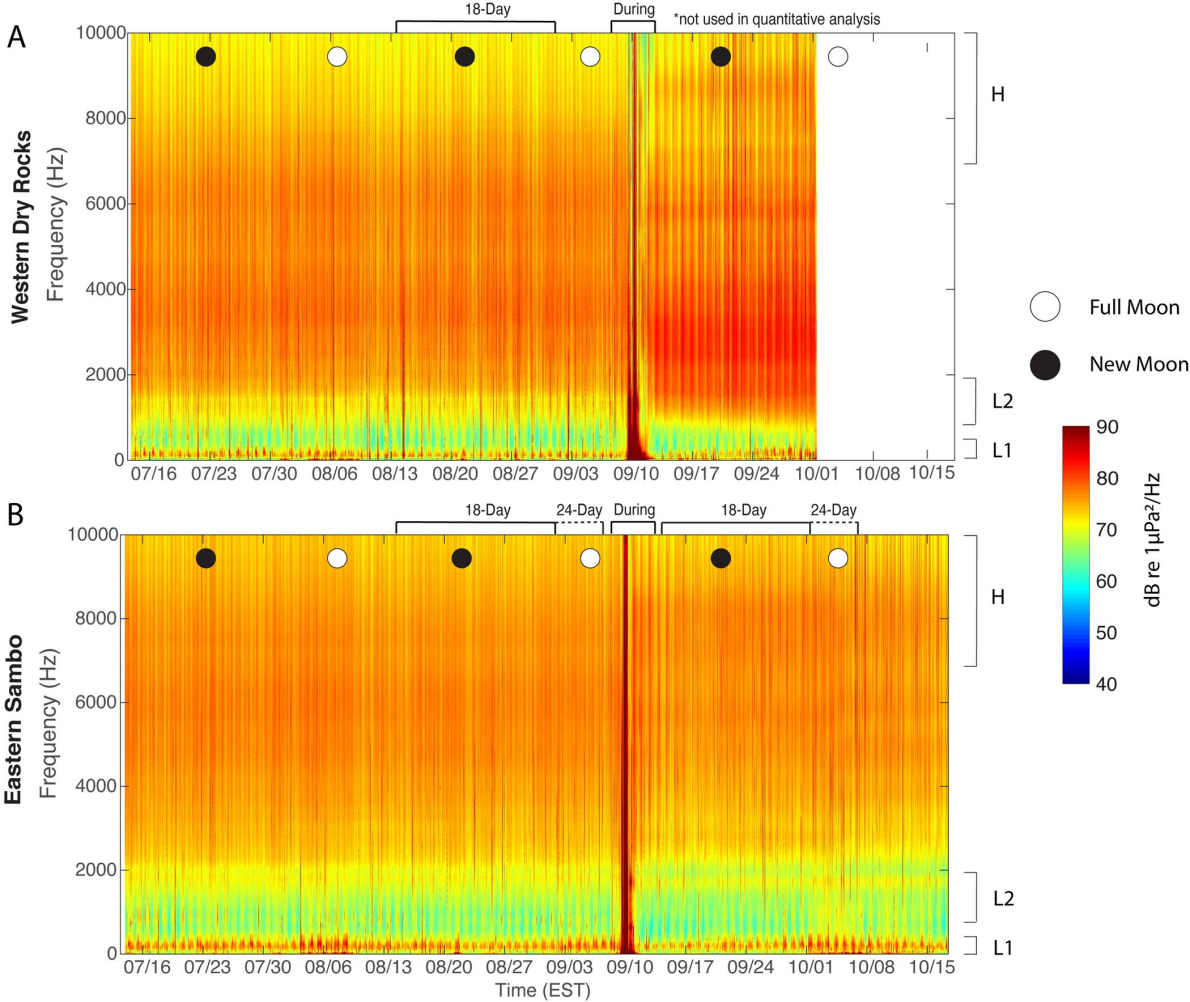
Deployment period spectrogram. Spectrogram displaying the power spectral density (PSD in dB re 1μPa^2^/Hz) for Western Dry Rocks (A) and Eastern Sambo (B). Frequency bands are denoted as follows: H, high frequency (7,000–20,000Hz); L1 low frequency (50-300Hz); L2 low frequency (1,200–1,800Hz). Open and filled circles indicate the full and new moons respectively. Hurricane Irma made landfall on September 10, 2017. Data within the WDR post-window was not valid or used for quantitative analysis. Spectrogram was generated from the average spectra within each two-minute recording (NFFT = 2^14^, (Δ*f*) = 2.93 Hz).

**Fig 6:**
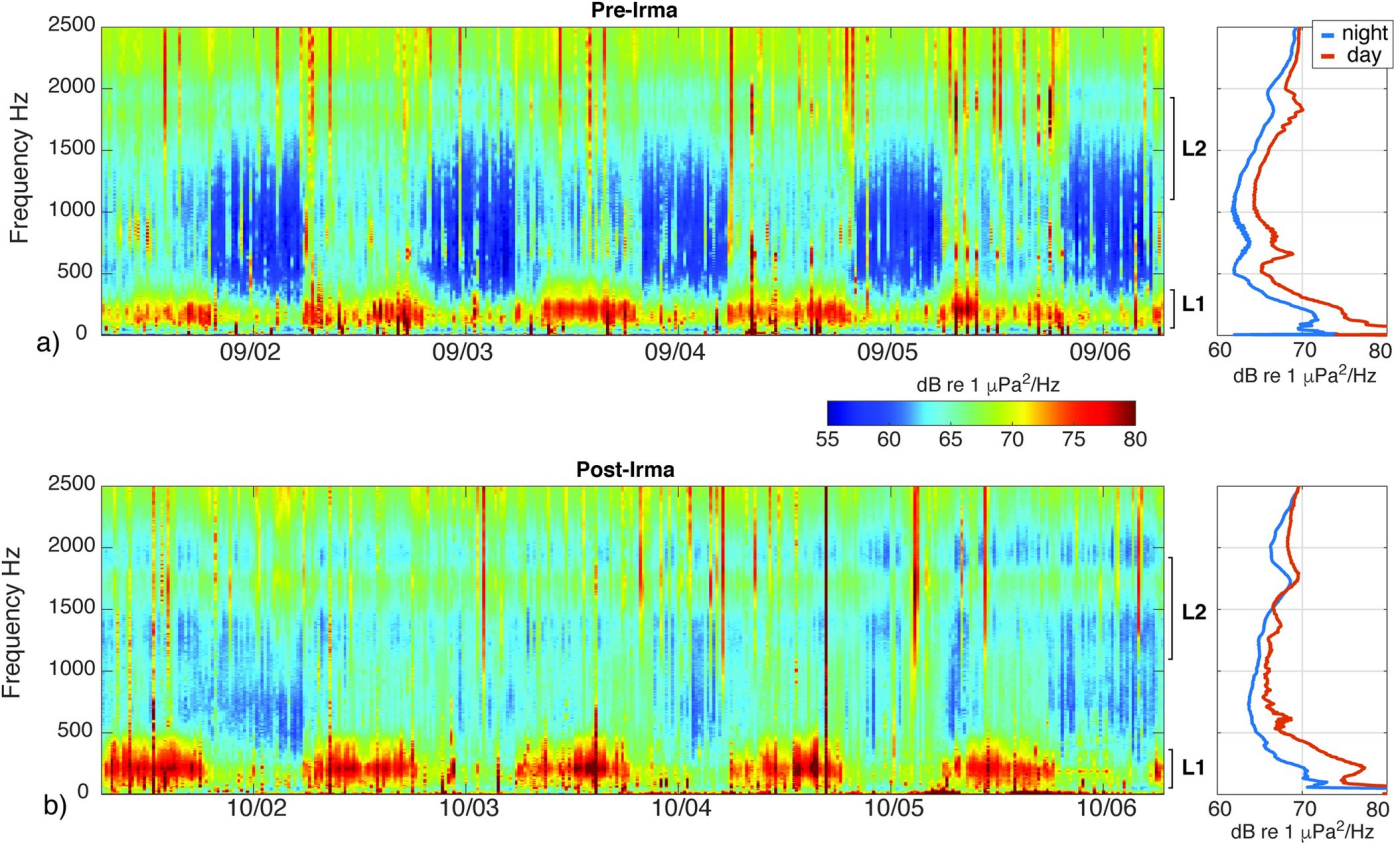
Short-duration spectrograms from Eastern Sambo. Spectrograms displaying the low frequency patterns of sound production during 5-day windows around the full moons that occurred (a) before and (b) after the passage of Hurricane Irma. Spectrograms are derived using the average spectra with each two minute recording. Time-axis ticks indicate midnight EST. Sound pressure levels are elevated during daytime hours, relative to the nighttime hours. The daily pattern of sound production reflects the acoustic activity and/or presence of multiple species (see call example in S1 Fig). The diurnal pattern in low-frequency (L1) sound production is present before and after the storm. The diurnal pattern of mid-frequency (L2) sound production is a less pronounced, and appears to weaken after the passage of the storm. Panels on the right show average sound pressure levels during daytime and nighttime recordings averaged over the 5-day windows.

Both sites showed temporal patterns in the biophony evident with their long-term spectrograms (Figs 5 and 6). A daily pattern of fish vocalizations within the L1 frequency band was apparent at WDR and ESB over the ~2 month recording period before the hurricane, with increased sound levels around the full moons in August and September (Fig 5). Fish calls within both low frequency bands were masked or absent during the storm, before reappearing immediately after the storm (Figs 5 and 6). The apparent post-storm shift in high-frequency sound levels at WDR likely reflected a change in sensitivity of the hydrophone after it became detached from the mooring (Fig 5A). The low frequency bands at ESB diminished in intensity during the waning part of the lunar cycle and became more pronounced approaching the October full moon (Fig 5B). Additionally, the less pronounced, yet persistent fish calling evident in the L2 band was observed before and after the storm at ESB (Figs 5B and 6). The L2 band captured broadband fish calls, including the upper range of pulsated “grunts” (>1000Hz) and aggregated “knocks” between 1200-2500Hz, as well as including the lower range of snapping shrimp sound production in the high frequency band. Snapping shrimp activity within the H band persisted before and after the storm at both sites (Fig 5).

### Temporal soundscape patterns

**Fig 7:**
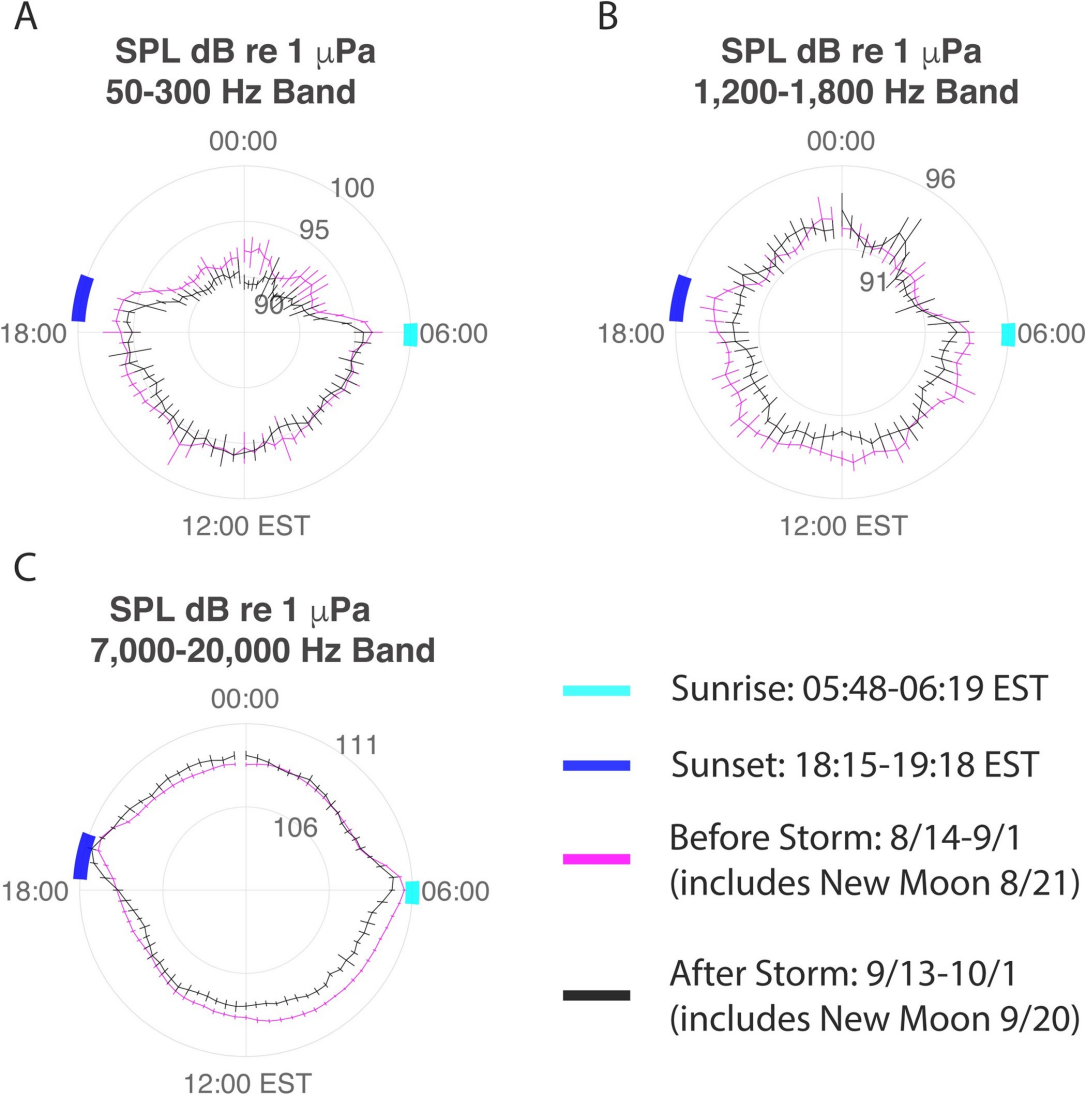
Polar diagram for Eastern Sambo. Polar diagram of Sound Pressure Levels (SPLs) for ESB for the 18-day observation window before (magenta) and after (black) the hurricane. Means for each recording interval are shown with 3-point moving average. Error bars represent the 68% confidence interval of mean. Data are displayed for A) L1 frequency band (50-300Hz); B) L2 frequency band (1,200–1,800Hz); and C) H frequency band (7,000–20,000Hz). Local sunrise (05:48–06:19 EST) and sunset (18:15–19:18 EST) times during the deployment are shown in cyan and blue, respectively.

The daily patterns in SPLs before and after the storm were examined for the ESB site. Within the three frequency bands, trimmed means were calculated for each recording interval (00:00, 00:20… 23:40) over the 18- and 24-day windows capturing the same portion of the lunar cycle before and after the storm. The results for the 18-day windows are displayed in Fig 7, along with their bootstrapped confidence intervals. The dominant temporal pattern was a diurnal rhythm (day vs. night) in sound production, along with a small increase in high frequency noise during crepuscular periods. The daily pattern of low and high frequency sound production was largely maintained after the storm, with only small shifts in the average loudness. Within the L1 band, a small decrease in the average SPL is observed during the nighttime hours, with little change in the average level during the daytime hours. For the L2 band, a small decrease in the average SPL is observed during the daytime hours, with little change observed at night.

**Fig 8:**
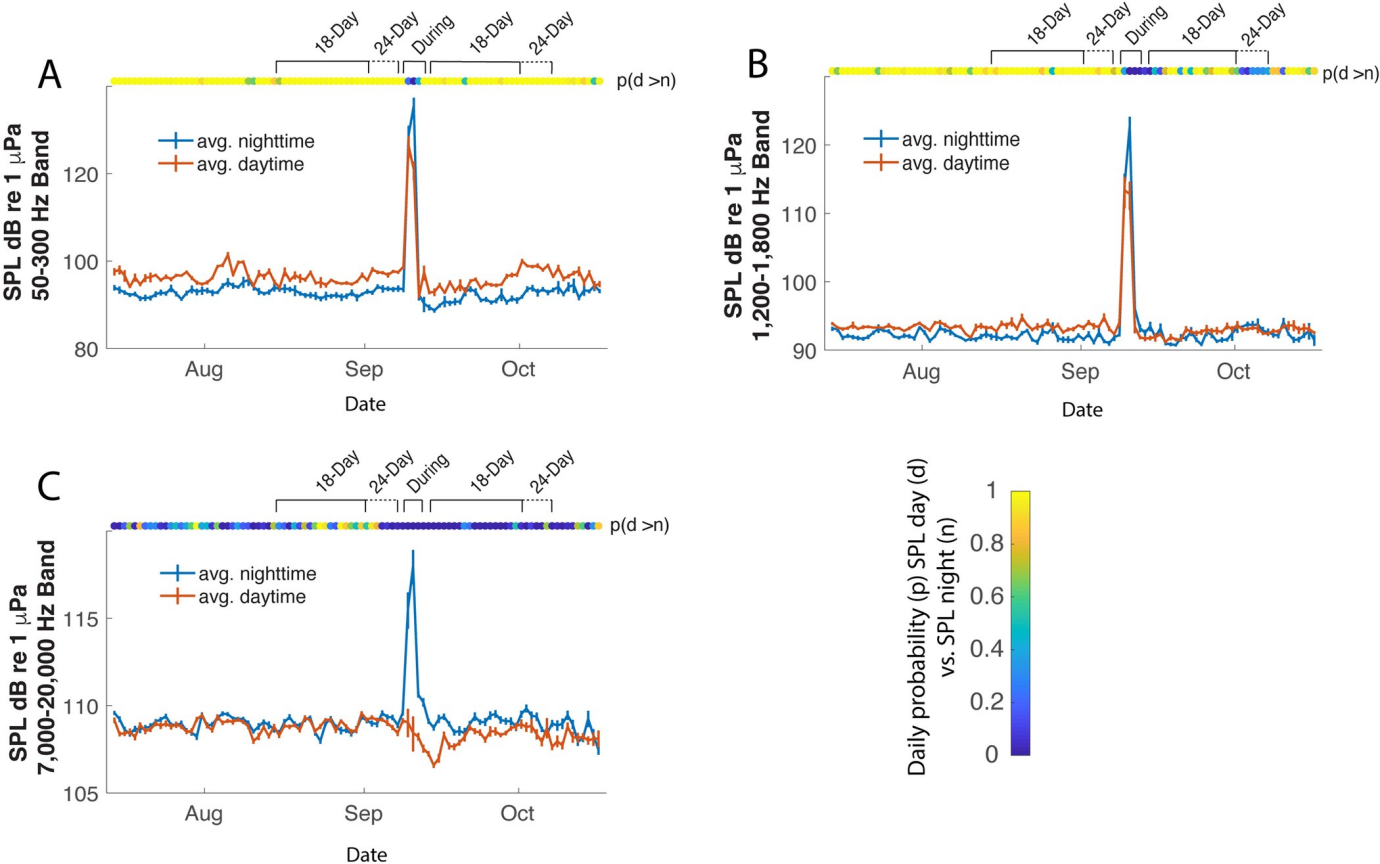
Eastern Sambo diurnal patterns. Mean daytime (red) and nighttime (blue) SPL during the deployment period July 14^th^–October 15^th^. Error bars represent the 68% confidence interval for the mean daytime and nighttime SPL within each 24-hour period. Data are displayed for the A) L1 frequency band (50-300Hz); B) L2 frequency band (1,200–1,800Hz); and C) H frequency band (7,000–20,000Hz). The color bar shows probability that daytime SPL is greater than nighttime SPL, *p* ≈ 1 indicates significantly higher daytime sound levels, and *p* ≈ 0 indicates significantly higher nighttime sound levels on a given day. Test periods are within an 18-day lunar cycle before the hurricane August 14^th^–September 1^st^ (includes August 21^st^ new moon) and after the hurricane September 13^th^–October 1^st^ (includes September 20^th^ new moon).

To investigate the diurnal patterns in more detail, the mean daytime and nighttime SPLs, along with their bootstrapped confidence intervals, were calculated daily for each frequency band (Fig 8). For each 24-hour period, the probability that the mean daytime SPL is greater than the mean nighttime SPL was estimated from the resampled means. Within the L1 frequency band, daytime SPL was consistently higher than nighttime SPL (*p* ≈ 1), except for the time window when the storm passed over the reef (Fig 8A). Within the L2 frequency band, daytime SPL was consistently higher than nighttime SPL (*p* ≈ 1) before the storm; however, there was no consistent diurnal pattern after the storm (Fig 8B). Within the H frequency band, there was no persistent diurnal pattern before the storm, yet daytime sound production decreased slightly after the storm creating a persistent pattern of higher nighttime SPL relative to daytime SPL (*p* ≈ 0) (Fig 8C).

**Fig 9:**
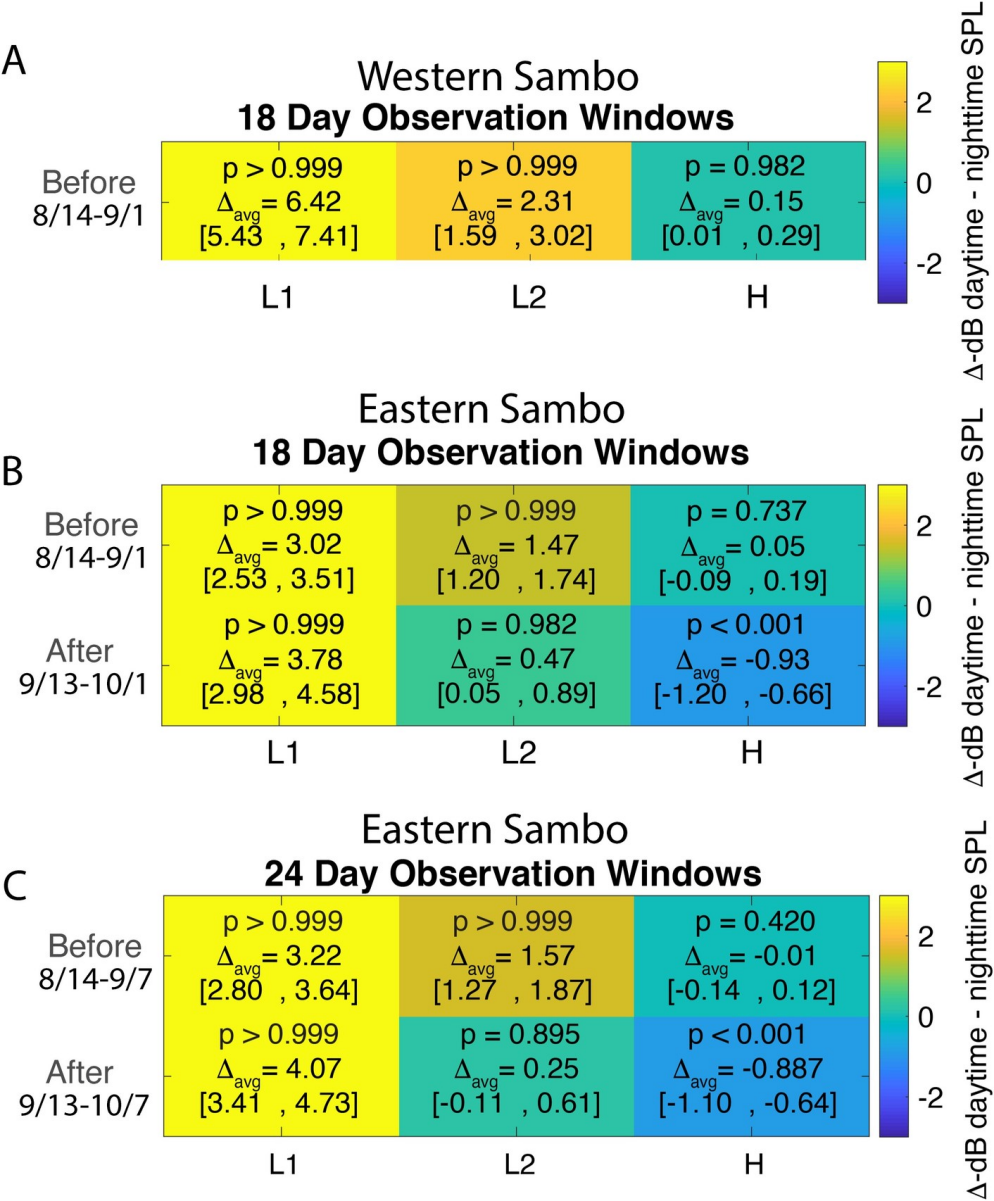
Pairwise bootstrap analysis results. Pairwise bootstrap (n = 5000) of mean differences, 95% confidence, and probabilities (p) daytime mean SPL > nighttime mean SPL for 18-day observation window at Western Dry Rocks (A) and Eastern Sambo (B). An additional pairwise analysis is given for Eastern Sambo for 24-day observation window (C). Frequency bands are denoted as follows: L1 low frequency (50-300Hz); L2 low frequency (1,200–1,800Hz); H, high frequency (7,000–20,000Hz). The color-bar represents the change in SPL (dB) between daytime-nighttime paired SPLs, with the 95% confidence range for decibel differences given in brackets. High p values and positive changes in decibel levels indicate periods when the average daytime SPL was higher than average nighttime SPL. Low p values and negative changes in decibel levels indicate periods when the average nighttime SPL was higher than average daytime SPL.

The magnitude and significance of these diurnal patterns in SPLs were quantified further by resampling the paired daytime and nighttime means over the 18- and 24-day windows before and after the storm. Fig 9 summarizes these results, reporting the mean diurnal difference (Δ_*avg*_) and its 95% confidence interval. Time windows with *p* ≈ 1, and positive confidence intervals, exhibited significantly higher daytime SPL, relative to nighttime SPL; or conversely, time windows with *p* ≈ 0, and negative confidence intervals, exhibited significantly higher nighttime SPL, relative to daytime SPL.

Before the hurricane, daytime SPL over the 18-day window was higher than nighttime SPL in the L1 band at both WDR (Δ_*avg*_ = 6.42*dB*) and ESB (Δ_*avg*_ = 3.02*dB*), with *p* ≈ 1. This diurnal difference was maintained with similar amplitude at ESB after the storm (Δ_*avg*_ = 3.78*dB*, *p* ≈ 1). The same pattern of higher daytime SPL than nighttime SPL was observed over the 24-day windows. Within the L2 band, before the storm daytime SPL also was higher than nighttime SPL at both WDR (Δ_*avg*_ = 2.31*dB*) and ESB (Δ_*avg*_ = 1.47*dB*), with *p* ≈ 1. This pattern weakened slightly at ESB after the storm, within both the 18- (Δ_*avg*_ = 0.47*dB*) and 24-day (Δ_*avg*_ = 0.25*dB*) windows, *p* = ~0.98. Within the H band prior to the storm, a small diurnal difference was observed only at WDR (Δ_*avg*_ = 0.15*dB*, *p* = 0.98). After the storm, however, the nighttime SPLs were elevated slightly relative to daytime SPL within both the 18- (Δ_*avg*_ = -0.93*dB*) and 24-day (Δ_*avg*_ = -0.89*dB*) windows, *p* ≈ 0 at ESB.

## Discussion

This study used passive acoustics to characterize the impacts of a major hurricane on a coral reef soundscape and the underlying temporal changes within the biophony that reflect biological behavior and activity. Observing changes in temporal patterns at hourly and daily scales for both the high and low frequency band, representative of ecologically important soniferous taxa, provided evidence that coral reef soundscapes may be resilient to a natural, acute disturbance despite experiencing physically destructive conditions. The extent to which a coral reef soundscape recovers to pre-disturbance patterns in sound pressure levels may depend on known characteristics of resilience in coral reef ecosystems, such as structural complexity or relative abundance of herbivorous species [131 and references therein], as well as characteristics of the storm itself, such as wind-speed, direction and duration.

### The influence of abiotic factors on the underwater soundscape during a disturbance

The expanse of the hurricane wind swath (64kt radius) exposed both sites (WDR and ESB) to high levels of acoustic energy, yet the potential of inflated exposure estimates from WDR’s hydrophone as it detached and presumably came into contact with fast moving sediment and debris did not allow for uncertainty estimates. Spectral densities during the hurricane did increase and produced narrow, wave-like peaks at high frequencies that may be explained by hydrodynamic processes. For example, previous studies indicate air-sea interactions can generate bubble formations that vary with wind speed intensity [132, 133], and bubble formation and dissipation can be produced via wave action [134, 135]. Moreover, air-sea interactions and the resulting swell of waves can vary depending on water depth and the structural complexity of the reef as current velocities can change when interacting with physical features. As previously described, WDR is characterized by relatively wide spur-and-groove channels, whereas ESB is dominated by a matrix of massive reef building corals and micro-patch reefs with relatively low sloping sand channels. Down-welling current velocities can intensify along the reef slope (spur) and increase vertically over the grooves of spur-and-groove reef formations [136]. Physical attributes of reef formations also drive other hydrodynamic processes such as refraction, dissipation, and shoaling—attributes that dictate the force and momentum of flow within the water column [137]. Therefore, the difference in sound spectral densities at each site during the storm passage may relate to stronger circulatory flows of wave action funneling into deep groove channels at WDR compared to ESB. Environmental changes during the hurricane, such as a decrease in bottom temperature and barometric pressure, may have had minimal impacts on the biophony as the presence of resident soniferous species immediately following the storm provides an alternative perspective on pre-storm migration patterns of fish and sharks seen in related studies [138, 139].

### Hurricane impacts to coral reef soundscapes

There are very few examples of how coral reef soundscapes respond to hurricane impacts, and of those, there is little quantitative information on specific impacts to soniferous reef fish groups. In contrast with previous coral reef soundscape studies that observe temporal patterns in the low frequency band across a wide frequency range (e.g. 0-3kHz), our results partitioned the low band to distinguish between reef fish families such Serranidae, Holocentridae, and Pomacentridae (represented by the L1 frequency band), and Haemulidae, Lutjanidae, Scaridae, and Sciaenidae (represented by the L2 frequency band). Nonetheless, the increased SPL at low frequencies during the daytime, relative to the nighttime, can likely be viewed as the integrated signature of multiple soniferous species with varying abundances and acoustic behaviors.

Reef fish chorusing around lunar phases were more prominent at ESB than WDR, and the presence of both the L1 and L2 frequency bands suggest the presence of a range of fish families during the same lunar phase despite impacts from Hurricane Irma. Fish chorusing was sometimes indicative of spawning behavior, and essential spawning locations are commonly populated by multiple species [140–143]. In a related study, Hurricane Charley (category 4) passed directly over Charlotte Harbor, Florida, USA yet nightly fish chorusing during spawning events yielded louder SPLs during and a few days after the hurricane than before, suggesting fish distribution patterns or behavior may not have been impacted by the hurricane [144]. Although the magnitude of change in low frequency-band sound pressure levels within the Irma observation windows (18- and 24-days) tested in this study were not significant, increased spatial coverage of soundscape characterization within a site using multiple hydrophones may have revealed different results.

The magnitude of change in diurnal sound pressure levels varied for each frequency band across the observation windows in this study. The L1 band at ESB was most resilient to change as average daytime sound levels maintained louder sound levels than paired nighttime sound levels during both observation windows. The L2 band also followed a similar diurnal trend as the L1 band at both sites; however, diurnal patterns in ESB’s L2 band weakened post-hurricane due to a decrease in daytime SPLs. This result differs from observations within the coral reefs Puerto Rico, where nighttime chorusing is reported to have increased following the passage of Hurricanes Irma and Maria [145, 146].

Diel migrations and nocturnal activity documented by acoustic tagging (telemetry), represented by the L2 band in this study, has been observed for grunts [147] and snappers [148, 149]. These species typically form mixed-species schools in refuge space underneath reef outcroppings or ledges during daylight hours before migrating to forage on adjacent seagrass beds around dusk [150–153]. The reductions in habitat quality (e.g. habitat degradation, turbidity) following a hurricane may have provided enhanced opportunities for cryptic or nocturnal species to forage or find mates during low visibility conditions and presumably low predation risk, which could promote a relatively broad range of vocalizations among reef fish [146, 154].

Diurnal snapping shrimp activity, as characterized by the H-frequency band, appeared resilient to the hurricane disturbance, with little change in snapping shrimp activity in the weeks following the storm. The H band at ESB did not show any significant difference between day-night SPLs before Irma, with only a small (~0.2 dB) difference developed in the weeks after the storms as daytime SPL decreased slightly. Recent studies in Puerto Rico revealed snapping shrimp inhabiting a shallow reef were silenced or masked during Hurricane Maria and did not return to crepuscular peaks in sound production until several days after hurricane passage [145, 146].

In this study, the coral reef soundscape post-Irma reflected the response of both fish and invertebrate behavior (e.g. daily, diurnal chorusing patterns) to a large-episodic disturbance in the form of a Category 4 Hurricane. Temporal patterns in the biophony at ESB appeared resilient to the acoustic energy exposure, change in environmental variables, and physical damage caused by Hurricane Irma. There are very few studies of how the soundscape of an ecosystem responds to a major environmental disturbance. Gasc et al. [52] highlighted changes in acoustic composition of an isolated desert after a wildfire event in which not only was acoustic activity diminished at burned sites, but the soundscape also reflected a change in taxonomic species distribution (e.g. insects, birds) and vegetative response (e.g. floral regeneration) post-disturbance. Their results are supported by traditional disturbance ecology studies where the resulting ecosystem reflected the severity of the disturbance and revealed which biological legacies (i.e. taxa-specific traits of survivors, remaining habitat structure) contribute to the re-establishment of an ecosystem [15, 155, 156].

In conclusion, this study characterized environmental variables associated with the passage of a Category 4 hurricane on a coral reef, and the associated temporal patterns in the biophony before, during, and after a natural disturbance. The short-term response of Eastern Sambo’s coral reef soundscape appeared resilient to the acoustic energy exposure, change in environmental variables, and physical damage caused by Hurricane Irma. Underwater soundscapes can be a complimentary ecological tool useful in characterizing small, yet important shifts in ecological communities during disturbances with localized impacts.

## Supporting information

S1 Fig Fish call spectrograms. Representative waveforms (top) and spectrograms (bottom) for the L1 low frequency band 50-300Hz: (A) Serranid growl, (B) fish “chirps”; and the L2 low frequency band 1200-1800Hz: (C) Haemulid “grunts”, (D) rapid aggregated “knocks”. Mean amplitudes were calculated using a bandpass filter 30-3000Hz and a steepness of 0.65. Spectrograms were calculated using a window length of 2048Hz with 50% overlap.
(TIF)

S2 Fig Illustration of trimmed sound pressure level time series. S1 Illustration of the trimmed sound pressure level time series applied to a section of the pre-storm storm data from Eastern Sambo site. The red line in panel A) shows the original broadband time series generated by calculating the root-mean-square (rms) sound pressure level in each 2-minute recording collected every 20 minutes. The blue line shows the time series after eliminating those files with the largest 2% of the amplitudes during the combined pre- and post-storm window. These trimmed data were used in calculating daytime and nighttime means. The largest amplitude spikes removed by this process are associated with files that contain one or more fish bumps. These signals do not represent sound, but can have a major influence on the calculated sound pressure levels. For example, the sound pressure level of the file shown in panel B) has a value of 113 dB rms re 1 μPa when averaged over the first 90 seconds of the file; this is consistent with expected background noise levels. However, when the series of fish bumps are included in the calculation, the amplitude rises by more than 30 decibels. Panel C) shows an individual bump signal. These signals are often clustered temporally, but typically occur in no more than 1 or 2 files per day. The trimming of the time series also removes a handful of files (3–4 per week) containing the sounds of a nearby small boat, as shown in panel D). The resulting trimmed time series better represents the underlying diurnal pattern of acoustic noise with the environment and is used to assess patterns of biophony.
(TIF)
