# Correction: Hurricane impacts on a coral reef soundscape

**DOI:** 10.1371/journal.pone.0258456

**Published:** 2021-10-06

**Authors:** Kayelyn R. Simmons, David B. Eggleston, DelWayne R. Bohnenstiehl

There is an error in Fig 9A. The figure is titled “Western Sambo” and the correct name is “Western Dry Rocks”. The authors have provided a corrected version here.

**Fig 9:**
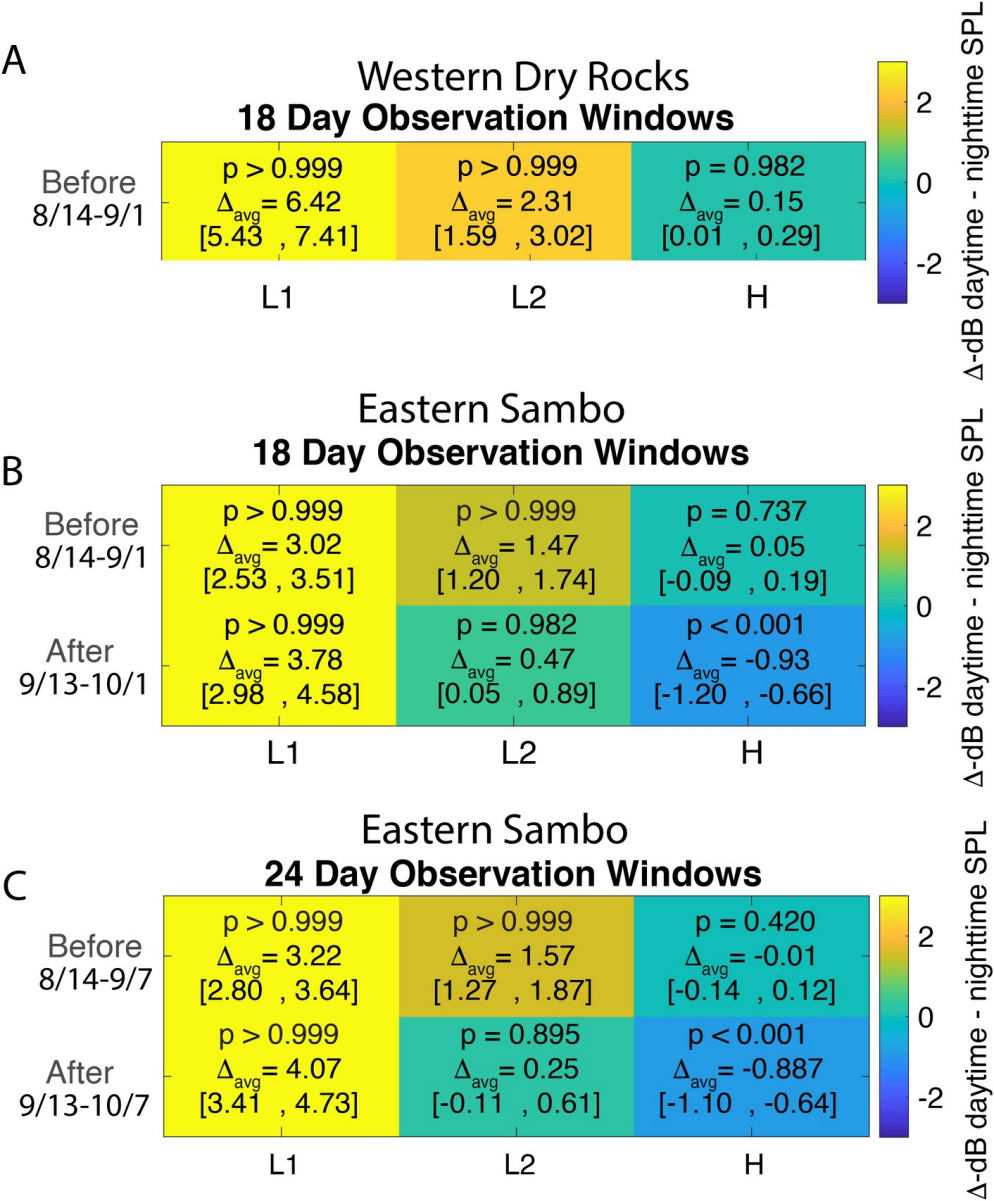
Pairwise bootstrap analysis results. Pairwise bootstrap (n = 5000) of mean differences, 95% confidence, and probabilities (p) daytime mean SPL > nighttime mean SPL for 18-day observation window at Western Dry Rocks (A) and Eastern Sambo (B). An additional pairwise analysis is given for Eastern Sambo for 24-day observation window (C). Frequency bands are denoted as follows: L1 low frequency (50-300Hz); L2 low frequency (1,200–1,800Hz); H, high frequency (7,000–20,000Hz). The color-bar represents the change in SPL (dB) between daytime-nighttime paired SPLs, with the 95% confidence range for decibel differences given in brackets. High p values and positive changes in decibel levels indicate periods when the average daytime SPL was higher than average nighttime SPL. Low p values and negative changes in decibel levels indicate periods when the average nighttime SPL was higher than average daytime SPL.
