# Supplementary figures and images for: Hurricane impacts on a coral reef soundscape
Source: PLoS One. 2021 Feb 24;16(2):e0244599. doi: 10.1371/journal.pone.0244599 (PMC7904201; doi:10.1371/journal.pone.0244599)

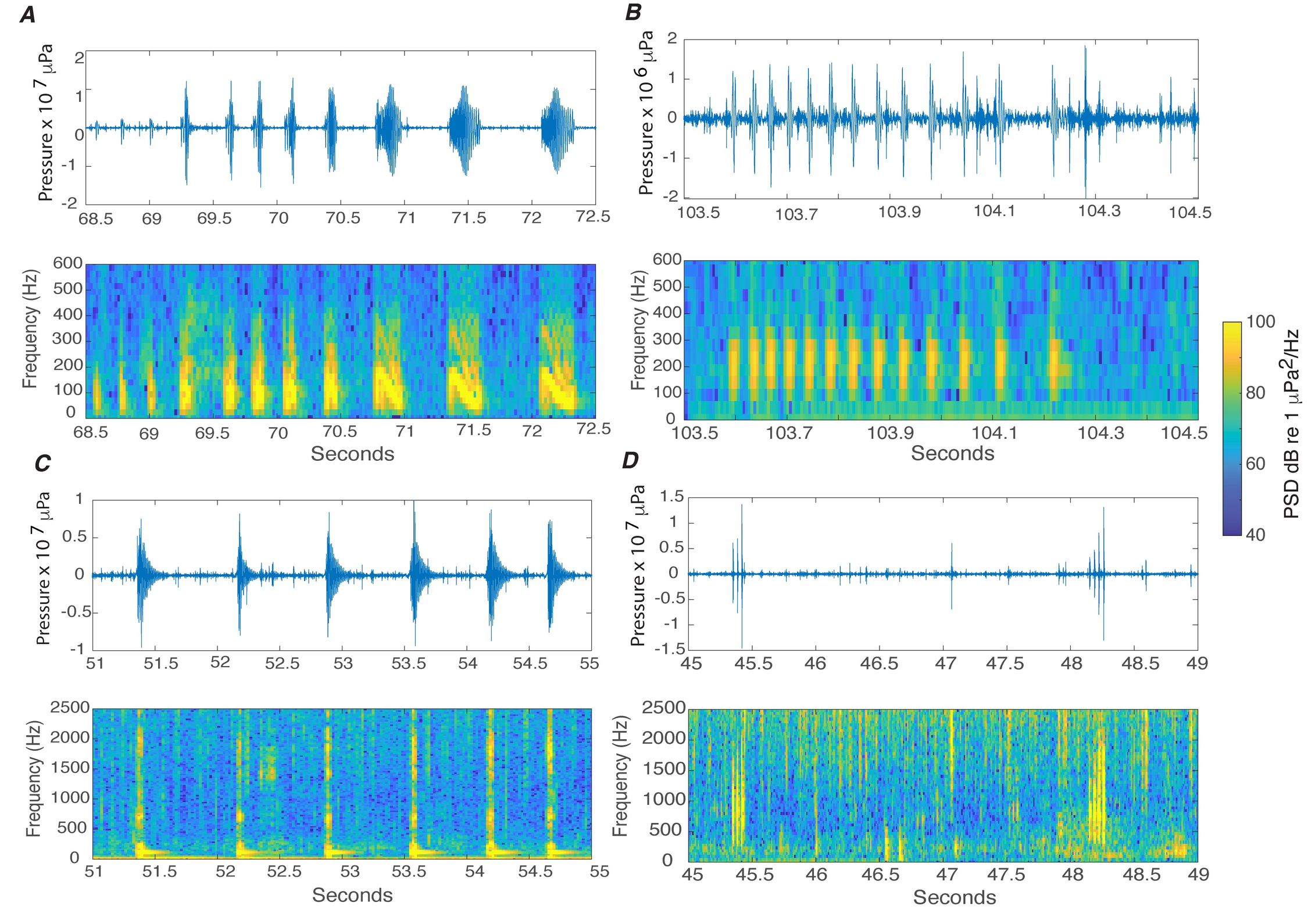

Supplement: S1 Fig — Representative waveforms (top) and spectrograms (bottom) for the L1 low frequency band 50-300Hz: (A) Serranid growl, (B) fish “chirps”; and the L2 low frequency band 1200-1800Hz: (C) Haemulid “grunts”, (D) rapid aggregated “knocks”. Mean amplitudes were calculated using a bandpass filter 30-3000Hz and a steepness of 0.65. Spectrograms were calculated using a window length of 2048Hz with 50% overlap. (TIF) [file pone.0244599.s001.tif]

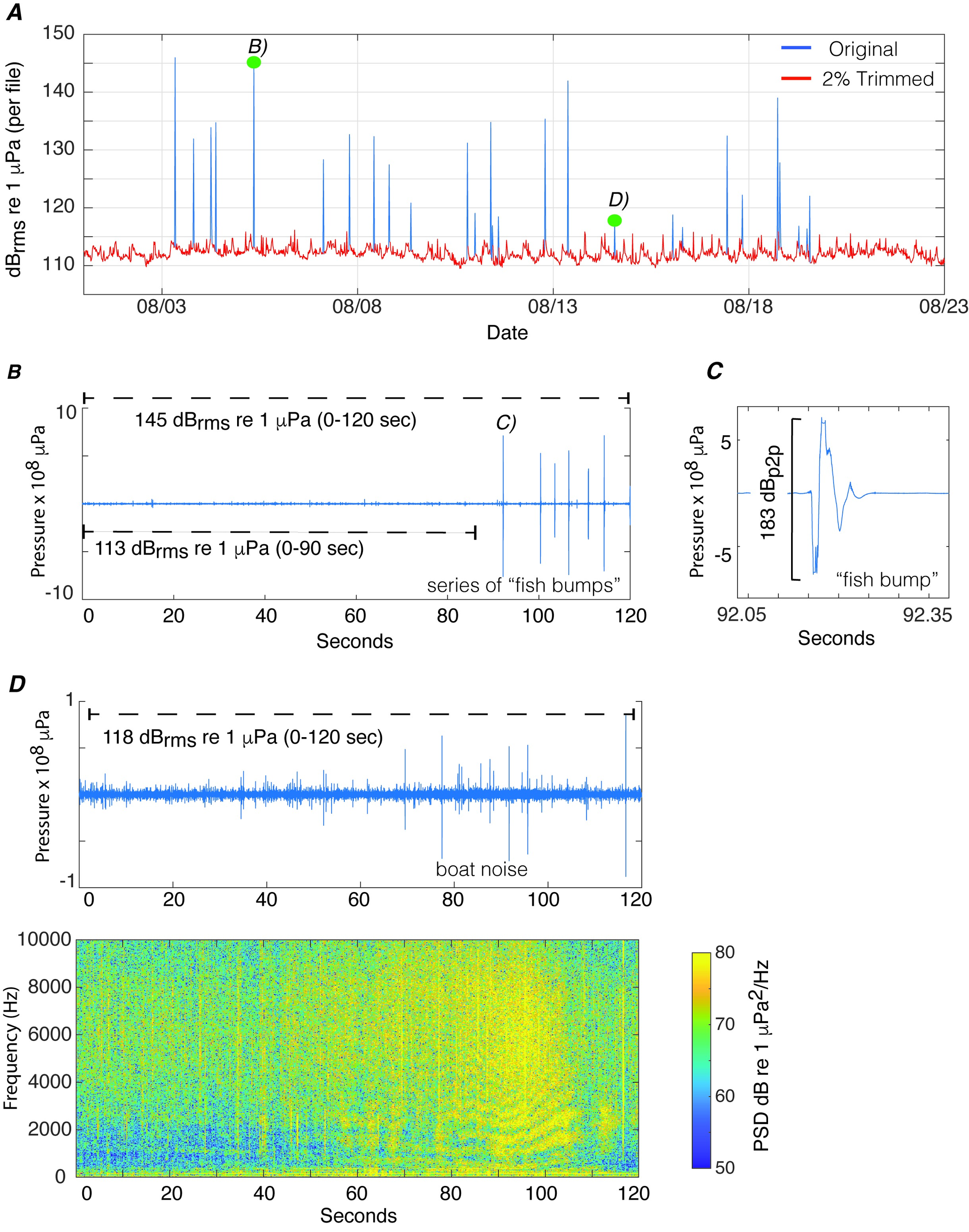

Supplement: S2 Fig — S1 Illustration of the trimmed sound pressure level time series applied to a section of the pre-storm storm data from Eastern Sambo site. The red line in panel A) shows the original broadband time series generated by calculating the root-mean-square (rms) sound pressure level in each 2-minute recording collected every 20 minutes. The blue line shows the time series after eliminating those files with the largest 2% of the amplitudes during the combined pre- and post-storm window. These trimmed data were used in calculating daytime and nighttime means. The largest amplitude spikes removed by this process are associated with files that contain one or more fish bumps. These signals do not represent sound, but can have a major influence on the calculated sound pressure levels. For example, the sound pressure level of the file shown in panel B) has a value of 113 dB rms re 1 μPa when averaged over the first 90 seconds of the file; this is consistent with expected background noise levels. However, when the series of fish bumps are included in the calculation, the amplitude rises by more than 30 decibels. Panel C) shows an individual bump signal. These signals are often clustered temporally, but typically occur in no more than 1 or 2 files per day. The trimming of the time series also removes a handful of files (3–4 per week) containing the sounds of a nearby small boat, as shown in panel D). The resulting trimmed time series better represents the underlying diurnal pattern of acoustic noise with the environment and is used to assess patterns of biophony. (TIF) [file pone.0244599.s002.tif]
